# Cell-Penetrating Mx1 Enhances Anti-Viral Resistance against Mucosal Influenza Viral Infection

**DOI:** 10.3390/v11020109

**Published:** 2019-01-28

**Authors:** Hi Eun Jung, Ji Eun Oh, Heung Kyu Lee

**Affiliations:** 1 Biomedical Science and Engineering Interdisciplinary Program, Korea Advanced Institute of Science and Technology (KAIST), Daejeon 34141, Korea; hieun.jung@kaist.ac.kr; 2 Graduate School of Medical Science and Engineering, KAIST, Daejeon 34141, Korea; jieun.oh@kaist.ac.kr; 3 KAIST Institute for Health Science and Technology, KAIST, Daejeon 34141, Korea

**Keywords:** Mx1, influenza virus, cell-penetrating peptide, type I interferon

## Abstract

Dynamin-like GTPase myxovirus resistance protein 1 (Mx1) is an intracellular anti-viral protein following the activation of type I and type III interferon signaling. Mx1 inhibits viral replication by blocking the transcription of viral RNA, and a deficiency in this protein enhances susceptibility to influenza infection. Thus, Mx1 could be another efficient target of anti-influenza therapy. To test our hypothesis, we fused poly-arginine cell-penetrating peptides to the C terminus of Mx1 (Mx1-9R) and examined the anti-viral activity of Mx1-9R in vitro and in vivo. Madin-Darby Canine Kidney epithelial cells internalized the Mx1-9R within 12 h. Pre-exposure Mx1-9R treatment inhibited viral replication and viral RNA expression in infected cells. Further, intranasal administration of Mx1-9R improved the survival of mice infected with the PR8 influenza viral strain. These data support the consideration of Mx1-9R as a novel therapeutic agent against mucosal influenza virus infection.

## 1. Introduction

Influenza-type virus is an *Orthomyxoviridae* virus that causes an acute febrile respiratory disease called influenza [1]. After entering the host through the nasal cavities, influenza virus infects bronchiolar–alveolar epithelial cells, macrophage, and dendritic cells (DCs) [2,3,4,5]. The virus then replicates and spreads throughout the airways. Pandemic influenza has occurred several times throughout the years, resulting in millions of deaths and unpredictably affecting public health [6]. Although many influenza vaccines have been developed to strengthen the protective immune responses in potential hosts, antigenic drift and antigenic shift cause viral variations that render the evolved virus only weakly affected by pre-existing vaccines [1,5,7,8]. Thus, an antigen-independent anti-viral agent provides the greatest hope for a lasting solution to influenza infection.

Type I interferons (IFNs) are important regulators in innate anti-viral responses. Protective immune responses are activated to eliminate pathogenic invaders following the recognition of pathogen-associated molecular patterns (PAMPs) via pattern recognition receptors (PRRs) on host cells [9]. Type I IFNs mediate various mechanisms that improve host protection, including interferon-stimulated gene (ISG) induction [10]. ISGs induce an anti-viral state in the host by restricting viral replication and disrupting viral genomes. Myxovirus resistance proteins (Mx proteins), intracellular anti-viral dynamin-like GTPases, are ISGs that inhibit influenza virus replication [5]. Mx proteins are highly conserved in most vertebrates. Human MxA exhibits about 67% amino acid sequence identity to mouse Mx1. Because Mx1 is an important intracellular regulator of viral replication, Mx1 deficiency increases susceptibility to influenza infection in mice [11,12]. The subcellular localization influences the anti-viral activity of Mx proteins. While rodent Mx1 is located in the nucleus and restricts nuclear viral replication, human MxA is located in the cytoplasm and restricts viral replication by preventing the nuclear import of viral RNA [11].

In this study, we combined murine Mx1 with a cell-penetrating protein to create an anti-viral agent effective against mucosal influenza infection. Arginine-rich cell-penetrating peptides (CPPs) [13] were fused with the C terminus of murine Mx1 to improve delivery efficacy, and the anti-viral activity of the resulting protein (i.e., Mx1-9R) was assessed in vitro and in vivo. Mx1-9R treatment inhibited viral replication and RNA expression in the infected cells, and treatment with Mx1-9R prior to influenza exposure increased murine resistance against influenza by restricting the viral propagation. Together, these results indicate that cell-penetrating Mx1 could be used as a therapeutic agent against mucosal influenza virus infection.

## 2. Materials and Methods

### 2.1. Construction of pET28a-Mx1-9R Vector

The *Escherichia coli* expression vector pET28a (a gift from HM Kim, Korea Advanced Institute of Science and Technology (KAIST), Daejeon, Korea) was used to express His-tagged fusion protein. To construct the insert DNA containing the 9 arginine-tagged mouse Mx1 (GenBank accession no: NM_010846, functional protein expressed in wild mouse strains) protein sequence, primers were designed as follows: NheI-Mx1, forward, 5′-CTAGCTAGCATGGATTCTGTGAATAATCTGTGCA-3′ (NheI site is underlined) and Mx1-9R-Not I, reverse, 5’-ATAAGAATGCGGCCGCCTAGCGGCGTCTGCGTCTGCGGCGTCTGCGATCGGAGAATTTGGCAAGCTTCTGCCGAGCCTC-3’ (NotI site is underlined). PrimeSTAR HS DNA polymerase (TAKARA, Shiga, Japan) was used to amplify the insert DNA. A 1954 bp-long Mx1-9R fragment was digested with NheI and NotI restriction enzymes (NEB, Ipswich, MA, USA) and inserted into the pET28a vector using T4 ligase (NEB). Cloned pET28a-Mx1 was amplified in DH5α chemically competent *E. coli* (Enzynomics, Daejeon, Korea) according to the manufacturer’s instructions. Then, BL21 (DE3) *E. coli* (Enzynomics) cells were transformed with the pET28a vector containing Mx1 fusion DNA sequence to express Mx1 fusion proteins.

### 2.2. Expression and Purification of Mx1-9R Fusion Protein

Transformed BL21 cells were cultured in Luria-Bertani (LB) broth (MB Cell, Los Angeles, CA, USA) containing 30 μg/mL of kanamycin (LPS solution, Daejeon, Korea) for 3 h at 37 °C, then incubated with 0.1 mM of Isopropyl β-D-1-thiogalactopyranoside (IPTG) (Goldbio, St. Louis, MO, USA) at 18 °C overnight. After IPTG induction, cells were centrifuged and resuspended in lysis buffer containing 50 mM of Tris-HCl (Welgene, Daegu, Korea), 0.5 M of NaCl (Welgene), and 10 mM of imidazole (Biosesang, Seongnam, Korea) with 1 mM of phenylmethylsulfonyl fluoride (Biobasic, Toronto, ON, Canada). Cell suspension was lysed by sonication for 40 × 10 s with 10 s intervals on ice. The lysate was centrifuged at 8000 rpm for 30 min at 4 °C, and His-tagged Mx1-9R was purified by Ni-NTA resin (Incospharm, Daejeon, Korea). Then, purified Mx1-9R fusion proteins were desalted using Amicon Ultra-15 Centrifugal Filter Units (Millipore Sigma, Darmstadt, Germany). Supernatant, pellet, flow through, and eluent were separated by 8% sodium dodecyl sulfate-polyacrylamide gel electrophoresis (SDS-PAGE), and the gel was stained with 0.05% Coomassie Blue R-250 (LPS solution). The amounts of recombinant proteins were quantified by Bradford assay or NanoDrop (Thermo Fisher, Waltham, MA, USA) measurements.

### 2.3. Construction, Expression, and Purification of OVA-9R Fusion Protein

To construct the pET28a-OVA-9R vector, insert DNA containing the 9 arginine-tagged chicken ovalbumin (GenBank accession no: NM_205152) was amplified with NheI-OVA forward primer (5’-CTAGCTAGCATGGGCTCCATCGGTGCAGCAAGCA-3’) and OVA-9R-NheI reverse primer (5’-ATAAGAATGCGGCCGCCTAGCGGCGTCTGCGTCTGCGGCGTCTGCGAGGGGAAACACATCTGCC-3’) using PrimeSTAR HS DNA polymerase (Takara). After amplification, pET28a-OVA-9R vector was constructed with NheI and NotI restriction enzymes (NEB) and T4 ligase (NEB). Cloned OVA-9R vector was amplified using DH5α chemically competent *E. coli* (Enzynomics) and transformed into the competent cell of *E. coli* BL21 (Enzynomics). To induce the recombinant OVA-9R protein, BL21 cells were cultured in LB broth containing 30 μg/mL of kanamycin for 3 h at 37 °C, then incubated with 0.1 mM of IPTG at 18 °C overnight. OVA-9R in inclusion bodies were solubilized using 6 M GuHCl containing 50 mM Tris (pH 8.0) buffer, and recombinant OVA-9R proteins were isolated by Ni-NTA resin. After purification, OVA-9R proteins were dialyzed four times for refolding. Briefly, isolated proteins were inoculated into the Slide-A-Lyzer™ Dialysis Cassettes (Thermo Fisher) with an exclusion size of 10 kDa, and dialyzed with 0.25 M of l-arginine, 200 mM of NaCl, 2 mM of reduced glutathione, and 0.5 mM of oxidized l-glutathione containing 50 mM Tris (pH 8.0) buffer at 4 °C overnight. Then, buffers were changed with 50 mM of l-arginine, 200 mM of NaCl, and 5% glycerol containing 50 mM Tris (pH 8.0) buffer, and dialyses were continued overnight. Next day, dialyses were performed with 200 mM NaCl and 5% glycerol containing 50 mM Tris (pH 8.0) buffer for 4 h at 4 °C. Refolded OVA-9R fusion proteins were desalted using Amicon Ultra-15 Centrifugal Filter Units (Millipore Sigma), and the amounts of recombinant proteins were quantified by NanoDrop (Thermo Fisher, Waltham, MA, USA) measurements.

### 2.4. Transduction of Mx1-9R Fusion Protein and Cell Viability Assay

To test whether Mx1-9R fusion proteins were internalized into cells, 4 × 10^4^ cells of Madin-Darby Canine Kidney (MDCK) epithelial cells were incubated with 50 μg/mL of Mx1-9R fusion proteins for 3 or 12 h. After incubation, cells were incubated with 5 μg/mL Alexa Fluor™ 633-conjugated wheat germ agglutinin (WGA) (Thermo Fisher) for 10 min at 37 °C to stain the plasma membrane. Then, cells were washed with pre-warmed Hanks’ balanced salt solution (HBSS; Welgene) and fixed with a solution of 4% paraformaldehyde in Dulbecco’s Phosphate Buffered Saline (DPBS; Welgene), and permeabilized with 0.2% Triton-X100 in DPBS. After blocking with 1% bovine serum albumin (BSA) and 3% goat serum in DPBS, internalized Mx1 fusion proteins in MDCK cells were stained with anti-Mx1 antibody (Santa Cruz, Dallas, TX, USA) for 2 h at room temperature (RT). Cells were washed three times with DPBS for 10 min and incubated with Cy3-conjugated conjugated goat anti-mouse IgG (Jackson Immunoresearch, West Grove, PA, USA) for 45 min at RT. Cover slips were mounted using Fluoroshield mounting medium with DAPI (Abcam, Cambridge, UK), and intracellular Mx1-9R proteins were detected using an LSM800 (Carl Zeiss, Oberkochen, Germany).

### 2.5. Cell Viability Assay

MDCK cells were seeded at 1 × 10^4^ cells/well in a 96-well plate and cultured for 16 h at 37 °C. After the cells reached confluence, 25–100 μg/mL Mx1-9R protein was added into cells and cultured for 24 h. Cell viability was measured using an EZ-cytox Cell Viability Assay Kit (DoGen, Seoul, Korea) for 30 min at 37 °C, and then cell viability was determined as the absorbance at 450 nm.

To test whether endotoxin contamination of Mx1-9R elicited an anti-viral state in cells, bone marrow cells were isolated from tibias and femurs of C57BL/6 mice. After red blood cell lysis using ammonium-chloride-potassium lysis buffer, 1 × 10^6^ cells were incubated with 25–100 μg/mL Mx1-9R proteins for 18 h and IFN-β and IL-1β levels in culture supernatants were measured by ELISA (eBioscience, San Diego, CA, USA).

### 2.6. Anti-Viral Activity of Mx1-9R in Vitro

To test the anti-viral activity of the Mx1-9R fusion proteins, 3 × 10^5^ MDCK cells were cultured with 50–125 μg/mL of Mx1-9R for 12 h before infection. Then, the cells were incubated with 0.01 Multiplicity of infection (MOI) of influenza type A PR8 (gifted from Akiko Iwasaki, Yale University, New Haven, CT, USA) virus for 48 h. To see whether post-infection Mx1-9R treatment could control the viral load in PR8-infected cells, 3 × 10^5^ MDCK cells were infected with 0.01 MOI of PR8 for 3 h. Then, 0.1–10 μg/mL of Mx1-9R was inoculated into the cells, and the cells were incubated for 48 h. Viral titers in culture supernatants were determined by plaque assay, and vRNA expression in the cells was assessed by real-time quantitative polymerase chain reaction (RT-qPCR).

### 2.7. RT-qPCR

Relative levels of vRNA were analyzed by RT-qPCR. Briefly, total RNA was isolated from PR8-infected MDCK cells using RNeasy Plus Mini Kit (Qiagen, San Francisco, CA, USA). The synthesis of cDNA from the RNA template was performed using oligo DT primers and M-MLV reverse transcriptase (Nobel Bio, Suwon, Korea). RT-qPCR analysis was conducted using SYBR® Green Realtime PCR Master Mix (Toyobo), and the primers were as follows:

PR8-NP, forward, 5’-GATTGGTGGAATTGGACGAT-3’ and

PR8-NP, reverse, 5’- AGAGCACCATTCTCTCTATT-3’;

PR8-M1, forward, 5’-AAGACCAATCCTGTCACCTCTGA-3’ and

PR8-M1, reverse, 5’-CAAAGCGTCTACGCTGCAGTCC-3’; and

GAPDH, forward, 5’-AACATCATCCCTGCTTCCAC-3’ and

GAPDH, reverse, 5-GACCACCTGGTCCTCAGTGT-3’.

### 2.8. Influenza Virus Infection In Vivo

Male C57BL/6 mice were purchased from DBL (Chungbuk, Korea). Mice were housed in a specific pathogen-free facility of Korea Advanced Institute of Science and Technology (KAIST). All animal care and procedures were approved by and performed according to the standards of the Institutional Animal Care and Use Committee (IACUC) of KAIST (KA2013-55, October, 24, 2013).

To assess the anti-viral effect of Mx1-9R fusion proteins, mice were anesthetized with an intraperitoneal injection of a ketamine/xylazine mixture. Then, 10 μg of Mx1-9R fusion proteins were intranasally administered to mice one day prior to the day of infection. For intranasal infection, ketamine (100 mg/kg) and xylazine (5.83 mg/kg) were injected intraperitoneally into mice to produce 30–60 min of anesthesia. After anesthesia, mice were infected intranasally with 20 μL of PR8 virus (25 PFU in phosphate-buffered saline (PBS)), and body weight loss and survival were monitored. Survival was estimated according to the Kaplan–Meier method, and significance was calculated by the log-rank test. Mice were euthanized when they lost more than 25% of their initial body weight. At 8 days post-infection, flu-infected mice were sacrificed, and bronchoalveolar lavage (BAL) fluids were collected by washing the trachea and lungs with 1 mL of phosphate-buffered saline (PBS).

### 2.9. Flow Cytometry

Intracellular staining and flow cytometry were performed to determine the effect of Mx1-9R on anti-viral immune responses in PR8-infected mice.

Mice were euthanized with carbon dioxide gas at 8 days post-infection. Lungs were minced and digested for 30 min at 37 °C in 1% fetal bovine serum (FBS) in Dulbecco’s Modified Eagle Medium (DMEM) with DNase I (Roche, Basel, Switzerland) and collagenase 4 (Worthington Biochemical Corporation, Lakewood, NJ, USA). Then, cells were centrifuged at 1500 rpm for 5 min at 4 °C and treated with HBSS buffer (Welgene) containing 5 mM of ethylenediaminetetraacetic acid and 5% of FBS for 5 min at 37 °C. Single cells were suspended using a 70-μm cell strainer and plunger. Lung cells were isolated by Percoll (GE Healthcare Life Sciences, Marlborough, MA, USA) density gradient centrifugation. After washing with PBS, single cells were harvested and treated with ammonium-chloride-potassium lysis buffer to eliminate red blood cells.

For pentamer staining, isolated cells were stained with FITC anti-mouse CD44 (Tonbo Bioscience, San Diego, CA, USA), PE anti-mouse NP_366–374_ Pentamer (Proimmune, Oxford, UK), PerCpCy5.5 anti-mouse CD4 (Tonbo Biosciences), PECy7 anti-mouse CD3e (eBioscience), APC anti-mouse CD11b (Biolegend, San Diego, CA, USA), APCCy7 anti-mouse CD8a (Tonbo Biosciences, USA), Alexa fluor 700 anti-mouse CD45.2 (Biolegend) antibodies, and DAPI.

For intracellular staining, isolated cells were stimulated with 10 μg/mL of NP_311-325_ (Peptron, Daejeon, Korea) peptides with 0.134 μL of GolgiStop, and 0.1 μL of GolgiPlug (BD Biosciences) for 13 h. Then, cells were stained with FITC anti-mouse CD44 (Tonbo Biosciences), APC anti-mouse IFN-γ (Biolegend), PECy7 anti-mouse CD3e (eBioscience), APCCy7 anti-mouse CD8a (Tonbo Biosciences), Alexa fluor 700 anti-mouse CD4 (Biolegend), violetFluor™ 450 anti-mouse CD45 (Tonbo Biosciences), and Brilliant Violet 510™ anti-mouse CD11b (Biolegend) antibodies. After staining, cells were analyzed on a FACS Fortessa (BD Biosciences).

### 2.10. Plaque Assay

MDCK cells were cultured at 5 × 10^5^ cells/well in complete DMEM media (Welgene), in a 6-well plate, overnight at 37 °C. After cells reached confluence, 250 μL of serial dilutions of culture supernatants from flu-infected cells or BAL collections of infected mice were added to the well and incubated at 37 °C for 1 h with shaking every 20 min. Then, plates were washed twice with PBS, and 2 mL/well 0.6% agarose medium were added to each well. After the gel became hard, the plates were cultured at 37 °C for 40 h.

### 2.11. Statistics

All data are presented as the mean ± standard error of the mean (SEM). Statistical significance was determined by Student’s *t*-test and two-way ANOVA analysis of variance with Bonferroni post-tests.

## 3. Results

### 3.1. Generation of Cell-Penetrating Mx1-9R Fusion Proteins

**Figure 1:**
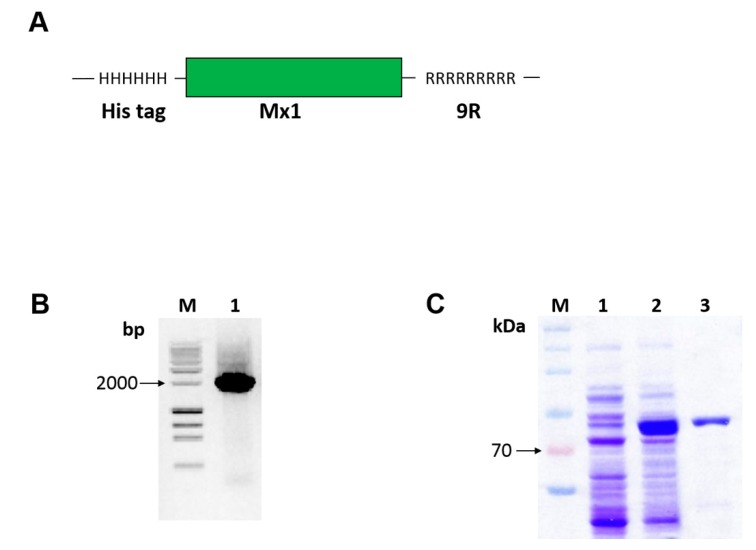
Construction, expression, and purification of cell-penetrating Mx1-9R. *Escherichia coli* cells were transformed with pET28a vector containing Mx1-9R fusion DNA sequence. After isopropyl β-D-1-thiogalactopyranoside (IPTG) induction, expressed Mx1-9R were purified by Ni-NTA column. (**A**) Structure of the Mx1-9R fusion protein was designed as described in the Methods. (**B**) Agarose gel electrophoresis of amplified Mx1-9R insert. (**C**) SDS-PAGE (2%) was performed to confirm the induction of Mx1-9R. BL21 (DE3). Lane 1: Whole lysates of control cells cultured without IPTG; lane 2: Whole cells induced with IPTG; lane 3: Purified Mx1-9R using Ni-NTA column.

To fuse the murine Mx1 with PTD 9-arginine sequence (see Figure 1A), we amplified insert DNA using PCR (see Figure 1B) and cloned the fusion Mx1-9R insert into the pET28a expression vector. Plasmid constructs were transformed into competent *E. coli* BL21, and the recombinant Mx1-9R fusion proteins were successfully induced with 0.1 mM of IPTG (see Figure 1C, lane 2) compared with noninduced control (see Figure 1C, lane 1). Next, Mx1-9R was purified by Ni-NTA resin, and buffer was exchanged using an Amicon centrifugal filter. SDS-PAGE results (see Figure 1C, lane 3) indicated that Mx1-9R was synthesized in a prokaryotic expression system.

### 3.2. Transduction of Mx1-9R Fusion Proteins

**Figure 2:**
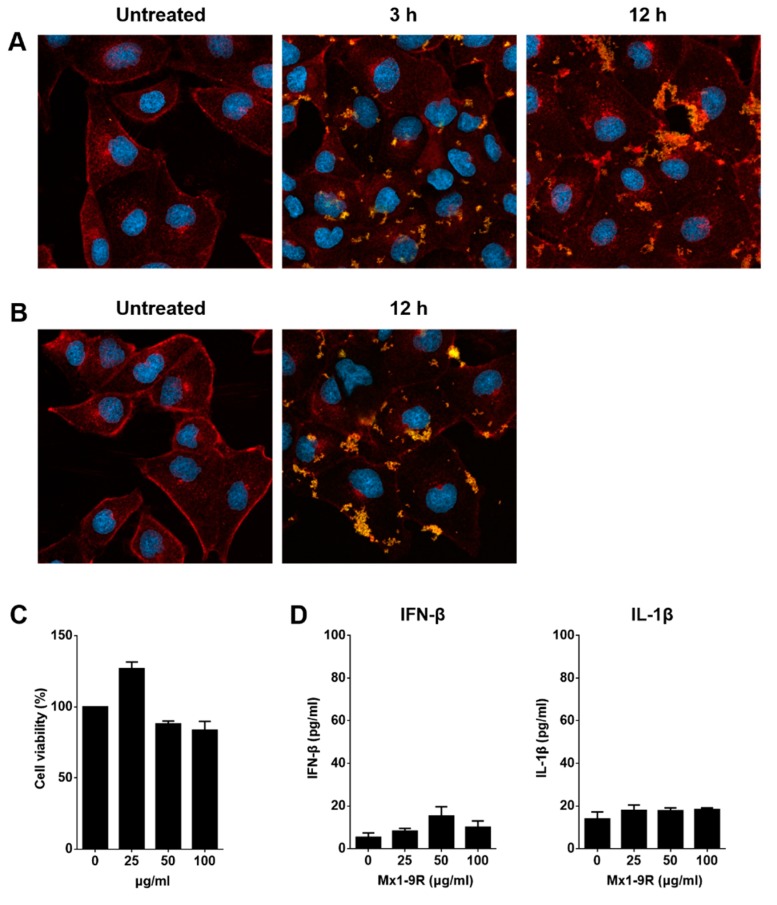
Transduction of Mx1-9R in vitro. Madin-Darby Canine Kidney (MDCK) cells were incubated with (**A**) 50 μg/mL or (**B**) 25 μg/mL of Mx1-9R. At 3–12 h after incubation, cells were permeabilized and stained with anti-Mx1 antibody, then internalization of Mx1 fusion proteins in MDCK cells was detected by confocal microscopy. (**C**) Cytotoxicity of Mx1-9R was determined by cell viability assay. MDCK cells were incubated with various concentrations of Mx1-9R for 24 h, and cell viability was determined by WST assay (*n* = 2–3). (**D**) Mouse bone marrow cells were cultured with Mx1-9R for 18 h and IFN-β and IL-1β levels in culture supernatants were measured by ELISA (*n* = 3).

To see whether Mx1-9R fusion proteins were transduced into cells, MDCK cells were cultured with 50 μg/mL of Mx1-9R for 3 and 12 h, and internalized proteins were detected by confocal microscopy. As shown in Figure 2A, Mx1-9R efficiently penetrated into cells within 3 h and accumulated in a time-dependent manner, and 25 μg/mL of Mx1-9 proteins efficiently penetrated into the cells further (Figure 2B). These results indicated that recombinant Mx1-9R sufficiently internalized into the cells.

Next, we tested the cytotoxicity of Mx1-9R. MDCK cells were incubated with 25–100 μg/mL of Mx1-9R for 24 h, and cell viability was determined by a Water-soluble tetrazolium salt (WST)-based assay. As shown in Figure 2C, Mx1-9R showed no significant cytotoxicity up to a concentration of 100 μg/mL. Then, we tested whether Mx1-9R induced type I IFN or IL-1β production from mouse bone marrow cells because endotoxin contamination affects the anti-viral state. Bone marrow cells were cultured with 25–100 μg/mL of Mx1-9R for 18 h, and IFN-β and IL-1β levels in culture supernatants were measured by ELISA. As shown in Figure 2D, Mx1-9R induced IFN-β, and IL-1β displayed very low expression levels. In conclusion, while endotoxin contamination induced type I IFN and IL-1β, the expression levels were too low to elicit an anti-viral state in cells.

### 3.3. Anti-Viral Activity of Mx1-9R Fusion Proteins in MDCK Cells

**Figure 3:**
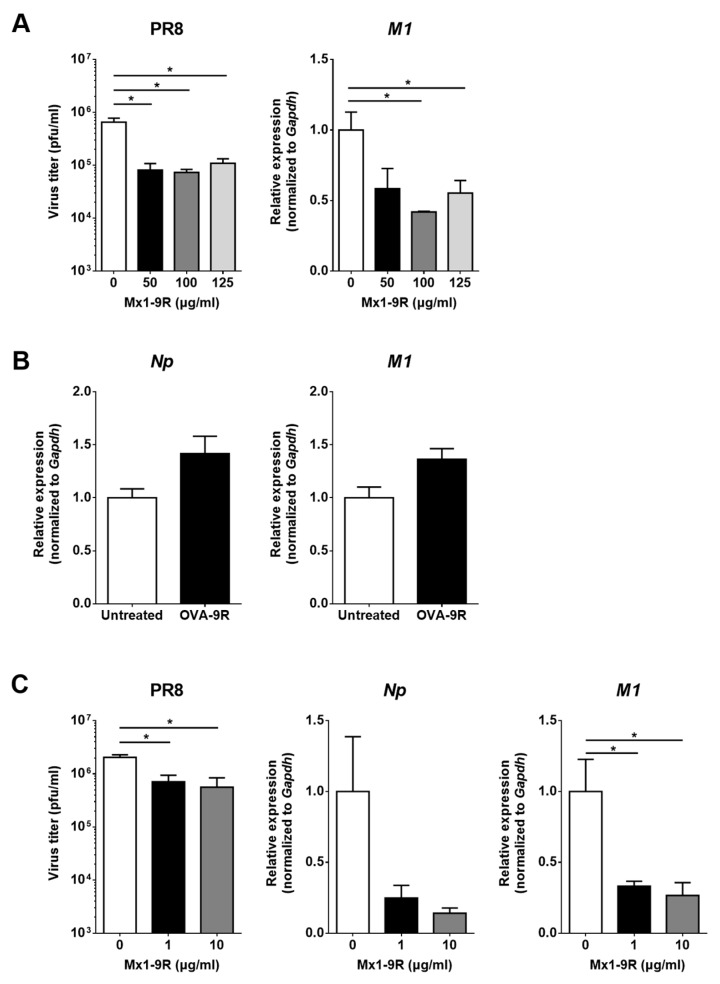
Mx1-9R inhibited influenza virus replication in infected cells. Mx1-9R was introduced into the MDCK cells (**A**) 12 h prior to or (**C**) 3 h post PR8 infection. Viral titers in the culture supernatant were determined by plaque assay, and relative levels of vRNA were analyzed by RT-qPCR (*n* = 3). Data are presented as the means ± SEM. (**B**) Recombinant 9 arginine conjugated ovalbumin (OVA-9R) was introduced into the MDCK cells 12 h prior to PR8 infection. Then, cells were infected with 0.01 MOI of PR8 for 24 h, and relative levels of vRNA were analyzed by RT-qPCR (*n* = 3). Data are presented as the means ± SEM.

The therapeutic effect of Mx1-9R treatment prior to infection was assessed by viral titration in culture supernatant and RT-qPCR. Because the internalization of Mx1-9R started within 3 h post-treatment, MDCK cells were incubated with 50–125 μg/mL of Mx1-9R for 12 h before infection. Then, 0.01 MOI of PR8 virus was inoculated into the cells, and viral titers in culture supernatants were determined 48 h after infection. As shown in Figure 3A, pre-infection Mx1-9R treatment efficiently reduced the viral titer in PR8-infected MDCK-cell culture supernatant while recombinant 9 arginine conjugated ovalbumin (OVA-9R) proteins did not inhibit the viral replication of PR8 (Figure 3B). In addition, the expression of the PR8-M1 gene was significantly lower in Mx1-9R-treated MDCK cells. These results indicate that pre-infection Mx1-9R treatment restricted viral propagation in vitro.

To test whether post-exposure Mx1-9R treatment controlled viral propagation in vitro, MDCK cells were infected with 0.01 MOI of PR8 for 3 h and 1 or 10 μg/mL of Mx1-9R were added to the cells. At 48 h after infection, the viral titers in culture supernatant and viral gene expressions were assessed. Interestingly, the viral titers in culture supernatant were dramatically decreased following 1 μg/mL of Mx1-9R treatment, and the copy numbers of viral M1 gene were also reduced compared with untreated cells (see Figure 3C). The protective efficiency of Mx1-9R was increased in a concentration-dependent manner. The results showed that viral propagation and RNA expression in the infected cells were inhibited by post-infection Mx1-9R treatment.

### 3.4. Anti-Viral Activity of Mx1-9R in Vivo

**Figure 4:**
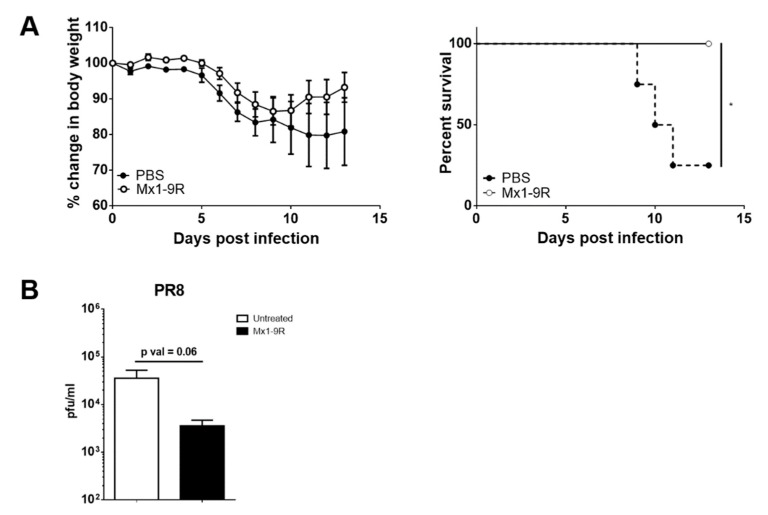

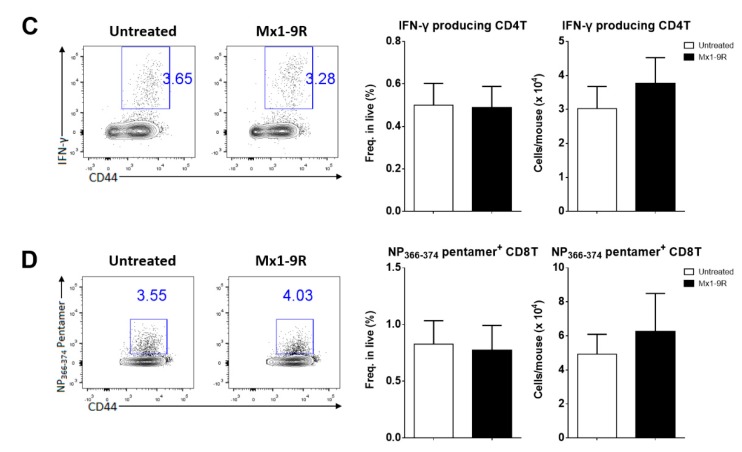
Protein transduction domain (PTD)-fused Mx1-9R improved the survival of influenza PR8-infected mice. Mx1-9R was intranasally administered to mice one day prior to the day of infection. Mice were infected by intranasal application of 25 PFU of PR8 virus. (**A**) Body weight loss and survival were measured according to the Kaplan–Meier method (*n* = 4–5). (**B**) Viral titers in BAL fluid were measured by plaque assay (*n* = 4–5). (**C**) Intracellular IFN-γ staining and (**D**) NP_366–374_ antigen-specific CD8 T-cell staining were performed to analyze the T-cell responses at 8 days post-infection (*n* = 4–6). Data are presented as the mean ± SEM.

Next, the anti-viral ability of Mx1-9R was tested in vivo. Mice were intranasally inoculated with 10 μg of Mx1-9R 1 day prior to the day of infection and infected with 25 PFU of PR8 virus; body weight change and survival of mice were then monitored. Although untreated control mice began to succumb to infection at day 9, Mx1-9R-treated mice displayed rapid recovery from body weight loss and demonstrated decreased mortality compared with the untreated control (see Figure 4A). We next measured the viral titers in the lungs of infected mice to test whether pre-infection Mx1-9R treatment reduced the viral burden. Flu-infected mice were sacrificed at 8 days post-infection, and BAL fluid viral titers were measured by plaque assay. As shown in Figure 4B, viral titers in the lungs were slightly lower in mice treated with Mx1-9R compared to the untreated group, though this trend did not reach statistical significance. These results demonstrate that pre-infection Mx1-9R treatment increased anti-viral resistance by regulating viral propagation in vivo.

Next, we hypothesized that Mx1-9R treatment induced effective T-cell responses to clear the infection. To assess the T-cell function, intracellular IFN-γ production was measured by flow cytometry. Lung cells were isolated from infected mice and restimulated with MHC class II restricted NP_311–325_ peptides. However, IFN-γ-producing CD4 T cells were comparable between groups (see Figure 4C). Further, Mx1-9R-treated mice demonstrated normal T-cell responses against influenza infection, and showed no significant changes in antigen-specific cytotoxic CD8 T cell populations in the lung compared to untreated mice (see Figure 4D). These results indicate that Mx1-9R treatment did not alter the protective T-cell responses against influenza virus, and adaptive immune responses were not crucial for the anti-viral activity of Mx1-9R.

## 4. Discussion

Mx1 is the first identified member of the Mx protein family, composed of GTPase-containing anti-viral effector proteins. Mx1 is induced by the activation of type I and type III interferon signaling pathways, and serves as an intracellular restriction factor against viral infection such as influenza [11]. Most inbred mouse strains (e.g., C57BL/6 and BALB/c) have nonfunctional mutant Mx1, and mice from these strains are highly susceptible to influenza infection [14,15,16,17,18]. The anti-viral functions of Mx1 have been investigated by in vitro and in vivo studies. For example, functional Mx1 transfection diminished the polymerase activity of influenza virus [19,20], and functional Mx1-carrying mice are highly resistant against pathogenic influenza infection such as pandemic 1918 and lethal human H5N1 [21,22], PR8 [23], and avian H5N2 Ab/Korea/ma81/07 virus [24]. Mx1 has been suggested as a key regulator of influenza virus, but whether Mx1 could be used as a therapeutic agent during influenza virus infection remains unclear.

CPPs, also known as protein transduction domains (PTDs), are short peptides that deliver large cargo molecules into cells without causing membrane damage, and are independent of receptors. In 1988, HIV trans-activator of transcription (TAT) protein was found to be rapidly taken up by cells. It was also capable of delivering large bioactive cargos into the cytoplasm [25,26,27]. Since then, various CPPs have been identified from natural sources (e.g., virus, *Drosophila*, and human), or have been designed artificially [28,29,30]. Poly-arginine is a cationic CPP commonly used in drug delivery systems. Previous researchers demonstrated that the cellular uptake efficacy of nona-arginine (9R) was superior to the HIV TAT [31,32]. Therefore, we used this 9R sequence to generate cell-penetrating murine Mx1 proteins.

Recent studies combined porcine Mx1 with PTDs to investigate the anti-viral activity of this PTD-poMx1 against vesicular stomatitis virus and classical swine fever virus (CSFV). They showed that PTD-poMx1 treatment suppressed virus proliferation in vitro [33,34]. They also demonstrated that PTD-poMx1 treatment successfully attenuated CSFV symptoms in CSFV-infected pigs in a dose-dependent manner [35]. However, whether these modified Mx1 proteins regulate influenza virus remained unclear. In this study, we generated cell-penetrating Mx1-9R fusion proteins and determined their antiviral activities in vitro and in vivo. Both pre- and post-infection Mx1-9R treatment inhibited viral replications in flu-infected MDCK cells, and Mx1-9R-treated mice showed significantly reduced mortality compared with untreated controls. However, we found no association between adaptive immune responses—which mediate viral clearance—and the superior protection against influenza virus in Mx1-9R-treated hosts. These results were consistent with previous research demonstrating high resistance to influenza virus in Mx1-carrying mice. The resistance was not due to adaptive immune responses [36]. Additionally, a recent study revealed that Mx1 inhibits influenza virus replication by inhibiting the interaction between PB2 and NP [12]. Further, MxA—the human homologue of Mx—oligomerizes around the viral nucleocapsid structure to inhibit the virus [37]. Therefore, the protective effect of cell-penetrating Mx1-9R might be mediated by the direct inhibition of viral ribonucleoprotein-complex assembly. Further investigation into the mechanisms that increase viral resistance in Mx1-PR-treated hosts should be conducted.

In conclusion, cell-penetrating Mx1-9R was efficiently internalized in cells within 12 h, and inhibited influenza viral propagation and RNA expression in infected cells. Indeed, pre-exposure treatment of Mx1-9R improved the anti-viral resistance of mice. These results reveal that our cell-penetrating Mx1 could be used as an effective therapeutic agent against mucosal influenza virus infection.
